# Measles — United States, January 1, 2020–March 28, 2024

**DOI:** 10.15585/mmwr.mm7314a1

**Published:** 2024-04-11

**Authors:** Adria D. Mathis, Kelley Raines, Nina B. Masters, Thomas D. Filardo, Gimin Kim, Stephen N. Crooke, Bettina Bankamp, Paul A. Rota, David E. Sugerman

**Affiliations:** 1 Division of Viral Diseases, National Center for Immunization and Respiratory Diseases, CDC.

**Summary**

**What is already known about this topic?**

Although endemic U.S. measles was declared eliminated in 2000, measles importations continue to occur. Prolonged outbreaks during 2019 threatened the U.S. measles elimination status.

**What is added by this report?**

During January 1, 2020–March 28, 2024, a total of 338 U.S. measles cases were reported; 29% of these cases occurred during the first quarter of 2024, almost all in persons who were unvaccinated or whose vaccination status was unknown. As of the end of 2023, U.S. measles elimination status was maintained.

**What are the implications for public health practice?**

Risk for widespread U.S. measles transmission remains low because of high population immunity. Enhanced efforts are needed to increase routine U.S. vaccination coverage, encourage vaccination before international travel, identify communities at risk for measles transmission, and rapidly investigate suspected measles cases to reduce cases and complications of measles.

## Abstract

Measles is a highly infectious febrile rash illness and was declared eliminated in the United States in 2000. However, measles importations continue to occur, and U.S. measles elimination status was threatened in 2019 as the result of two prolonged outbreaks among undervaccinated communities in New York and New York City. To assess U.S. measles elimination status after the 2019 outbreaks and to provide context to understand more recent increases in measles cases, CDC analyzed epidemiologic and laboratory surveillance data and the performance of the U.S. measles surveillance system after these outbreaks. During January 1, 2020–March 28, 2024, CDC was notified of 338 confirmed measles cases; 97 (29%) of these cases occurred during the first quarter of 2024, representing a more than seventeenfold increase over the mean number of cases reported during the first quarter of 2020–2023. Among the 338 reported cases, the median patient age was 3 years (range = 0–64 years); 309 (91%) patients were unvaccinated or had unknown vaccination status, and 336 case investigations included information on ≥80% of critical surveillance indicators. During 2020–2023, the longest transmission chain lasted 63 days. As of the end of 2023, because of the absence of sustained measles virus transmission for 12 consecutive months in the presence of a well-performing surveillance system, U.S. measles elimination status was maintained. Risk for widespread U.S. measles transmission remains low because of high population immunity. However, because of the increase in cases during the first quarter of 2024, additional activities are needed to increase U.S. routine measles, mumps, and rubella vaccination coverage, especially among close-knit and undervaccinated communities. These activities include encouraging vaccination before international travel and rapidly investigating suspected measles cases.

## Introduction

Measles is a highly infectious acute, febrile rash illness with a >90% secondary attack rate among susceptible contacts (*1*). High national 2-dose coverage with the measles, mumps, and rubella (MMR) vaccine led to the declaration of U.S. measles elimination* in 2000 (*2*). However, this elimination status was threatened in 2019 because of two prolonged outbreaks among undervaccinated communities in New York and New York City; these outbreaks accounted for 29% of all reported cases during 2001–2019 (*2*). To assess U.S. measles elimination status after the 2019 outbreaks and to provide context for understanding more recent increases in measles cases in 2024,^†^ CDC assessed the epidemiologic and laboratory-based surveillance of measles in the United States and the performance of the U.S. measles surveillance system during January 1, 2020–March 28, 2024.

## Methods

### Reporting and Classification of Measles Cases

Confirmed measles cases^§^ (*1*) are reported to CDC by state health departments through the National Notifiable Disease Surveillance System and directly (by email or telephone) to the National Center for Immunization and Respiratory Diseases. Measles cases are classified by the Council of State and Territorial Epidemiologists as import-associated if they were internationally imported, epidemiologically linked to an imported case, or had viral genetic evidence of an imported measles genotype (*1*); cases with no epidemiologic or virologic link to an imported case are classified as having an unknown source (*1*). For this analysis, unique sequences were defined as those differing by at least one nucleotide in the N-450 sequence (the 450 nucleotides encoding the carboxyl-terminal 150 nucleoprotein amino acids) based on the standard World Health Organization (WHO) recommendations for describing sequence variants^¶^ (*3*). Unvaccinated patients were classified as eligible for vaccination if they were not vaccinated according to Advisory Committee on Immunization Practices recommendations (*4*). A well-performing surveillance system was defined as one with ≥80% of cases meeting each of the following three criteria: classified as import-associated, reported with complete information on at least eight of 10 critical surveillance indicators (i.e., place of residence, sex, age, occurrence of fever and rash, date of rash onset, vaccination status, travel history, hospitalization, transmission setting, and whether the case was outbreak-related) (*5*), and laboratory-confirmed.

### Assessment of Chains of Transmission

Cases were classified into chains of transmission on the basis of known epidemiologic linkages: isolated (single) cases, two-case chains (two epidemiologically linked cases), and outbreaks (three or more epidemiologically linked cases). The potential for missed cases within two-case chains and outbreaks was assessed by measuring the interval between measles rash onset dates in each chain; chains with more than one maximum incubation period (21 days) between cases could indicate a missing case in the chain. This activity was reviewed by CDC, deemed not research, and was conducted consistent with applicable federal law and CDC policy.**

## Results

### Reported Measles Cases and Outbreaks

CDC was notified of 338 confirmed measles cases with rash onset during January 1, 2020–March 28, 2024 (Figure); cases occurred in 30 jurisdictions. During 2020, 12 of 13 cases preceded the commencement of COVID-19 mitigation efforts in March 2020. Among the 170 cases reported during 2021 and 2022, 133 (78%) were associated with distinct outbreaks: 47 (96%) of 49 cases in 2021 occurred among Afghan evacuees temporarily housed at U.S. military bases during Operation Allies Welcome, and 86 (71%) of 121 cases in 2022 were associated with an outbreak in central Ohio. During 2023, 28 (48%) of 58 cases were associated with four outbreaks. As of March 28, 2024, a total of 97 cases have been reported in 2024, representing 29% of all 338 measles cases reported during January 1, 2020–March 28, 2024, and more than a seventeenfold increase over the mean number of cases reported during the first quarter of 2020–2023 (five cases).

**FIGURE:**
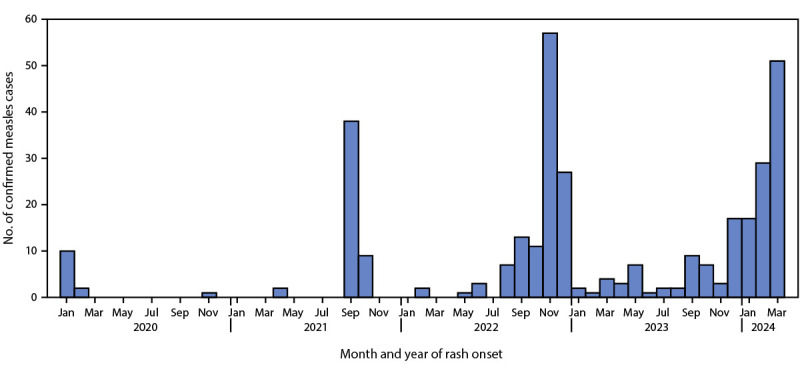
Confirmed measles cases, by month of rash onset (N = 338) — United States, January 1, 2020–March 28, 2024

### Characteristics of Reported Measles Cases

The median patient age was 3 years (range = 0–64 years); more than one half of cases (191; 58%) occurred in persons aged 16 months–19 years (Table). Overall, 309 (91%) patients were unvaccinated (68%) or had unknown vaccination status (23%); 29 (9%) had previously received ≥1 MMR vaccine dose. Among the 309 cases among unvaccinated persons or persons with unknown vaccination status, 259 (84%) patients were eligible for vaccination, 40 (13%) were aged 6–11 months and therefore not recommended for routine MMR vaccination, and 10 (3%) were ineligible for MMR because they were aged <6 months.^††^ Among 155 (46%) hospitalized measles patients, 109 (70%) cases occurred in persons aged <5 years; 142 (92%) hospitalized patients were unvaccinated or had unknown vaccination status. No measles-associated deaths were reported to CDC.

**TABLE:** Epidemiologic and laboratory characteristics of reported measles cases — United States, January 1, 2020–March 28, 2024

Characteristic	No. (%), by year						
	Total	2020	2021	2022	2023	2020–2023	2024
**Total measles cases**	**338**	**13**	**49**	**121**	**58**	**241**	**97**
**Age group**							
0–5 mos	**10 (3)**	0 (—)	5 (10)	0 (—)	0 (—)	5 (2)	5 (5)
6–11 mos	**57 (17)**	0 (—)	11 (22)	26 (21)	6 (10)	43 (18)	14 (14)
12–15 mos	**29 (9)**	1 (8)	5 (10)	13 (11)	3 (5)	22 (9)	7 (7)
16 mos–4 yrs	**106 (31)**	2 (15)	13 (27)	52 (43)	15 (26)	82 (34)	24 (25)
5–19 yrs	**85 (25)**	2 (15)	12 (24)	29 (24)	20 (34)	63 (26)	22 (23)
20–49 yrs	**47 (14)**	8 (62)	3 (6)	1 (1)	14 (24)	26 (11)	21 (22)
≥50 yrs	**4 (1)**	0 (—)	0 (—)	0 (—)	0 (—)	0 (—)	4 (4)
**Vaccination status**							
Unvaccinated	**230 (68)**	5 (38)	8 (16)	118 (98)	42 (72)	173 (72)	57 (59)
Unknown	**79 (23)**	5 (38)	40 (82)	1 (1)	10 (17)	56 (23)	23 (24)
Vaccinated, 1 dose	**17 (5)**	0 (—)	0 (—)	1 (1)	4 (7)	5 (2)	12 (12)
Vaccinated, 2 doses	**12 (4)**	3 (23)	1 (2)	1 (1)	2 (3)	7 (3)	5 (5)
**Import-associated cases**	**326 (96)**	12 (92)	48 (98)	121 (100)	53 (91)	234 (97)	60 (94)
International importation*	**93 (28)**	7 (54)	21 (43)	23 (19)	23 (40)	74 (31)	19 (20)
Import-linked^†^	**65 (19)**	4 (31)	18 (37)	11 (9)	19 (33)	52 (22)	13 (13)
Imported-virus or imported-virus–linked^§^	**168 (50)**	1 (8)	9 (18)	87 (72)	11 (19)	108 (45)	60 (62)
**Unknown source cases** ^¶^	**12 (4)**	1 (8)	1 (2)	0 (—)	5 (9)	7 (3)	5 (5)
Source WHO region for internationally imported cases**							
Eastern Mediterranean	**48 (52)**	3 (43)	21 (100)	9 (39)	4 (17)	37 (50)	11 (58)
African	**24 (26)**	0 (—)	0 (—)	18 (78)	5 (22)	23 (31)	1 (5)
European	**11 (12)**	1 (14)	0 (—)	0 (—)	7 (30)	8 (11)	3 (16)
South-East Asia	**11 (12)**	0 (—)	0 (—)	0 (—)	8 (35)	8 (11)	3 (16)
Americas	**2 (2)**	2 (29)	0 (—)	0 (—)	0 (—)	2 (3)	0 (—)
Western Pacific	**2 (2)**	1 (14)	0 (—)	0 (—)	0 (—)	1 (1)	1 (5)
**≥80% of 10 critical variables reported** ^††^	**336 (99)**	13 (100)	48 (98)	121 (100)	58 (100)	240 (100)	96 (99)
**Laboratory findings**							
Laboratory confirmed	**314 (93)**	13 (100)	48 (98)	109 (90)	53 (91)	223 (93)	91 (94)
IgM-positive only	**16 (5)**	2 (15)	0 (—)	4 (4)	5 (9)	11 (5)	5 (5)
rRT-PCR–positive^§§^	**298 (95)**	11 (85)	48 (100)	105 (96)	48 (91)	212 (95)	86 (95)
rRT-PCR–positive with genotyping completed	**221 (74)**	9 (82)	45 (94)	96 (91)	32 (67)	182 (86)	39 (45)
**No. of transmission chains**							
**Total**	**92**	**9**	**7**	**15**	**31**	**62**	**30**
Isolated cases^¶¶^	**62 (67)**	8 (89)	2 (29)	9 (60)	24 (77)	43 (69)	19 (63)
Two-case chains^¶¶^	**10 (11)**	0 (—)	2 (29)	1 (7)	3 (10)	6 (10)	4 (13)
Outbreaks (three or more cases)^¶¶^	**20 (22)**	1 (11)	3 (43)	5 (33)	4 (13)	13 (21)	7 (23)

**Abbreviations: **IgM = immunoglobulin M; rRT-PCR = real-time reverse transcription–polymerase chain reaction; WHO = World Health Organization.

* A case resulting from exposure to measles virus outside the United States as evidenced by at least some of the exposure period (7–21 days before rash onset) occurring outside the United States and rash onset occurring within 21 days of entering the United States without known exposure to measles during that time.

^†^ A case in a transmission chain epidemiologically linked to an internationally imported case.

^§^ A case for which an epidemiologic link to an internationally imported case was not identified, but for which viral sequence data indicate an imported measles genotype (i.e., a genotype that is not detected in the United States with a pattern indicative of endemic transmission).

^¶^ A case for which an epidemiologic or virologic link to importation or to endemic transmission within the United States cannot be established after a thorough investigation.

** Percentage is percentage of international importations. Four cases among persons who traveled to both the Eastern Mediterranean and African regions and one case in a person who traveled to both the Eastern Mediterranean and European regions were counted twice.

^††^ Place of residence, sex, age or date of birth, fever and rash, date of rash onset, vaccination status, travel history, hospitalization, transmission setting, and whether the case was outbreak related.

^§§^ Includes 65 cases among patients who received both positive rRT-PCR and positive IgM results.

^¶¶^ Percentage is percentage of total chains.

### Imported Measles Cases

Among all 338 cases, 326 (96%) were associated with an importation; 12 (4%) had an unknown source. Among the 326 import-associated cases, 200 (61%) occurred among U.S. residents who were eligible for vaccination but who were unvaccinated or whose vaccination status was unknown. Among 93 (28%) measles cases that were directly imported from other countries, 34 (37%) occurred in foreign visitors, and 59 (63%) occurred in U.S. residents, 53 (90%) of whom were eligible for vaccination but were unvaccinated or whose vaccination status was unknown. One (2%) case in a U.S. resident occurred in a person too young for vaccination, two (3%) in persons who had previously received 1 MMR vaccine dose, and three (5%) in persons who had previously received 2 MMR vaccine doses. The most common source for internationally imported cases during the study period were the Eastern Mediterranean (48) and African (24) WHO regions. During the first quarter of 2024, a total of six internationally imported cases were reported from the European and South-East Asia WHO regions, representing a 50% increase over the mean number of importations from these regions during 2020–2023 (mean of two importations per year from each region).

### Surveillance Quality Indicators

Overall, all but two of the 338 case investigations included information on ≥80% of the critical surveillance indicators; those two case investigations included information on 70% of critical surveillance indicators. Date of first case report to a health department was available for 219 (65%) case investigations; 127 (58%) cases were reported to health departments on or before the day of rash onset (IQR = 4 days before to 3 days after). Overall, 314 (93%) measles cases were laboratory confirmed, including 16 (5%) by immunoglobulin M (serologic) testing alone and 298 (95%) by real-time reverse transcription–polymerase chain reaction (rRT-PCR). Among 298 rRT-PCR–positive specimens, 221 (74%) were successfully genotyped: 177 (80%) were genotype B3, and 44 (20%) were genotype D8. Twenty-two distinct sequence identifiers (DSIds) (*3*) for genotype B3 and 13 DSIds for genotype D8 were detected (Supplementary Figure, https://stacks.cdc.gov/view/cdc/152776). The longest period of detection for any DSId was 15 weeks (DSId 8346).

### Chains of Transmission

The 338 measles cases were categorized into 92 transmission chains (Table); 62 (67%) were isolated cases, 10 (11%) were two-case chains, and 20 (22%) were outbreaks of three or more cases. Seven (35%) of 20 outbreaks occurred during 2024.^§§^ The median outbreak size was six cases (range = three–86 cases) and median duration of transmission was 20 days (range = 6–63 days). Among the 30 two-case chains and outbreaks, more than one maximum incubation period (21 days) did not elapse between any two cases.

## Discussion

Because of the absence of endemic measles virus transmission for 12 consecutive months in the presence of a well-performing surveillance system, as of the end of 2023, measles elimination has been maintained in the United States. U.S. measles elimination reduces the number of cases, deaths, and costs that would occur if endemic measles transmission were reestablished. Investigation of almost all U.S. measles cases reported since January 2020 were import-associated, included complete information on critical surveillance variables, were laboratory-confirmed by rRT-PCR, and underwent genotyping; these findings indicate that the U.S. measles surveillance system is performing well. A variety of transmission chain sizes were detected, including isolated cases, suggesting that sustained measles transmission would be rapidly detected. However, the rapid increase in the number of reported measles cases during the first quarter of 2024 represents a renewed threat to elimination.

Most measles importations were cases among persons traveling to and from countries in the Eastern Mediterranean and African WHO regions; these regions experienced the highest reported measles incidence among all WHO regions during 2021–2022 (*6*). During November 2022–October 2023, the number of countries reporting large or disruptive outbreaks increased by 123%, from 22 to 49. Global estimates suggest that first-dose measles vaccination coverage had declined from 86% in 2019 to 83% in 2022, leaving almost 22 million children aged <1 year susceptible to measles (*6*).

As has been the case in previous postelimination years (*7*), most imported measles cases occurred among unvaccinated U.S. residents. Increasing global measles incidence and decreasing vaccination coverage will increase the risk for importations into U.S. communities, as has been observed during the first quarter of 2024, further supporting CDC’s recommendation for persons to receive MMR vaccine before international travel (*4*).

Maintaining high national and local MMR vaccination coverage remains central to sustaining measles elimination. Risk for widespread U.S. measles transmission remains low because of high population immunity; however, national 2-dose MMR vaccination coverage has remained below the Healthy People 2030 target of 95% (the estimated population-level immunity necessary to prevent sustained measles transmission) (*8*) for 3 consecutive years, leaving approximately 250,000 kindergarten children susceptible to measles each year (*9*). Furthermore, 2-dose MMR vaccination coverage estimates in 12 states and the District of Columbia were <90%, and during the 2022–23 school year, exemption rates among kindergarten children exceeded 5% in 10 states (*9*). Clusters of unvaccinated persons placed communities at risk for large outbreaks, as occurred during the central Ohio outbreak in 2022: 94% of measles patients were unvaccinated and 42% were hospitalized (*10*). Monitoring MMR vaccination coverage at county and zip code levels could help public health agencies identify undervaccinated communities for targeted interventions to improve vaccination coverage while preparing for possible measles outbreaks. As of March 28, 2024, a total of 97 confirmed measles cases have been reported in the United States in 2024, compared with a mean of five cases during the first quarter of each year during 2020–2023. Similar to cases reported during 2020–2023, most cases reported during 2024 occurred among patients aged <20 years who were unvaccinated or whose vaccination status was unknown, and were associated with an importation. Rapid detection of cases, prompt implementation of control measures, and maintenance of high national measles vaccination coverage, including improving coverage in undervaccinated populations, is essential to preventing measles and its complications and to maintaining U.S. elimination status.

### Limitations

The findings in this report are subject to at least three limitations. First, importations might have been underreported: 4% of reported cases during the study period had no known source. Second, case investigations resulting in discarded measles cases (i.e., a diagnosis of measles excluded) are not nationally reportable, which limits the ability to directly evaluate the sensitivity of measles case investigations. However, surveillance remains sufficiently sensitive to detect isolated cases and outbreaks, and robust molecular epidemiology provides further evidence supporting the absence of sustained measles transmission in the United States. Finally, the date of first case report to a health department was not available for 35% of case investigations.

### Implications for Public Health Practice

The U.S. measles elimination status will continue to be threatened by global increases in measles incidence and decreases in global, national, and local measles vaccination coverage. Because of high population immunity, the risk of widespread measles transmission in the United States remains low; however, efforts are needed to increase routine MMR vaccination coverage, encourage vaccination before international travel, identify communities at risk for measles transmission, and rapidly investigate suspected measles cases to maintain elimination.

## Footnotes

* Elimination is defined as the absence of endemic measles virus transmission in a defined geographic area for ≥12 months in the presence of a well-performing surveillance system.

†
https://emergency.cdc.gov/han/2024/han00504.asp

§ A confirmed measles case was defined as an acute febrile rash illness with laboratory confirmation or direct epidemiologic linkage to a laboratory-confirmed case. Laboratory confirmation was defined as detection of measles virus–specific nucleic acid from a clinical specimen using real-time reverse transcription–polymerase chain reaction or a positive serologic test for measles immunoglobulin M antibody.

¶ Genotyping was performed at CDC and at the Vaccine Preventable Disease Reference Centers of the Association of Public Health Laboratories.

** 45 C.F.R. part 46.102(l)(2), 21 C.F.R. part 56; 42 U.S.C. Sect. 241(d); 5 U.S.C. Sect. 552a; 44 U.S.C. Sect. 3501 et seq.

†† MMR vaccine is not licensed for use in persons aged <6 months.

§§ At the time of this report, six measles outbreaks have ended, and one outbreak is ongoing. A measles outbreak is considered to be over when no new cases have been identified during two incubation periods (42 days) since the rash onset in the last outbreak-related case.
